# Supplementary material for: Street Food in Malaysia: What Are the Sodium Levels?
Source: Foods. 2022 Nov 24;11(23):3791. doi: 10.3390/foods11233791 (PMC9739403; doi:10.3390/foods11233791)
Supplement: Supplementary file 1 [file foods-11-03791-s001.zip › foods-1981593-supplementary.pdf]

FIGURE S1 : FLOWCHART OF DATA COLLECTION IN PHASE I AND PHASE II

Phase I (2020-2021)

Survey of street food in all 14 states of Malaysia

Determine the criteria of street food stalls based on definition by FAO (2007)

- Stalls located on streets and other similar public places that do not have a fixed building or are confined within four walls (including any individual stalls, stalls within the day and night markets).
- Ready-to-eat food and beverages made and/or sold by vendors and hawkers.

↓ Identify all the night and morning markets that were still operating for each state through the city councils' websites

Field survey of locally available street food stalls in all 14 states of Malaysia (N=10,520) using a survey form

- Selangor (n=2345)
- Kuala Lumpur (n=2146)
- Negeri Sembilan (n=820)
- Melaka (n=839)
- Johor (n=570)
- Kedah (n=217)
- Perlis (n=109)
- Perak (n=192)
- Penang (n=200)
- Terengganu (n=1395)
- Pahang (n=959)
- Kelantan (n=366)
- Sabah (n=137)
- Sarawak (n=243)

↓

Determine the total frequency for each street food surveyed (N=10,520) for each state

↓

Classify each street food surveyed (N=10,520) with the total frequency according to the recorded street food categories (main meals (MM), snacks (SN), and desserts (DS)) for each state

- Selangor (MM=792, SN=1000, DS=553)
- Kuala Lumpur (MM=883, SN=935, DS=328)
- Negeri Sembilan (MM=333, SN=305, DS=164)
- Melaka (MM=203, SN=480, DS=156)
- Johor (MM=207, SN=220, DS=143)
- Kedah (MM=118, SN=64, DS=35)
- Perlis (MM=42, SN=47, DS=20)
- Perak (MM=90, SN=65, DS=37)
- Penang (MM=113, SN=44, DS=43)
- Terengganu (MM=495, SN=504, DS=396)
- Pahang (MM=362, SN=356, DS=241)
- Kelantan (MM=92, SN=102, DS=172)
- Sabah (MM=54, SN=55, DS=28)
- Sarawak (MM=103, SN=57, DS=83)

↓

Identify and list out the top 15 most frequently available street foods among main meals, snacks, and desserts for each state

Phase II (2021-2022)
Food sampling and sodium analysis

Sampling of seven main meals, five snacks, and three desserts for each state (N=210) from the top 15 list

1. Purchase each type of street food from two different stalls within the respective states for sodium analysis.
2. Store the purchased street food in an ice box for transportation (5°C for 1.5 hours) to the laboratories.

↓

Preparation of street food samples for sodium analysis

1. Inspect the samples and remove any inedible portion such as bones.
2. Weigh each sample with the packaging still intact.
3. Blend together the same street food samples purchased from two different stalls.
4. Keep the homogenized samples in an airtight container and store in a -20℃ freezer, prior to its preparation for sodium analysis.

↓

Sodium analysis of street food samples

1. Weigh 1-3 g samples into a 50 ml polypropylene tube for wet digestion.
2. Add about 30 ml of 30% acid nitric into the tube.
3. Place the closed tube on a hot block at 95°C for 1.5 hours.
4. Prepare the intermediate standards of 0.1, 0.5, 1.0, 2.0, 5.0, 10, 20, 30, 50, and 100 ppm in 1L volumetric flasks with diluted nitric acid for calibration.
5. Analyze the sodium content in duplicates using inductively coupled plasma-optical emission spectrometry (Agilent Technologies, 5100 ICP-OES)

↓

Data analysis

TABLE S1 : STREET FOOD SURVEY FORM

SURVEY FRAME : _______________ **REGION >** **STATE >** **DISTRICT >** **CITY/AREA**

SURVEYOR NAME : DATE : TIME :

Objective: To determine the available street food distributions, categories and names, preparation method, and frequency in Malaysia.

Bear in mind during the survey, please ASK YOURSELF in order to keep in track, as well as taking PICTURE for food identification.

1. Why? - Refer street food definition and objectives.
2. What? - Category of street food (**main meals, dessert, snacks**). The specific common name.
3. How? - Preparation method [raw, semi-cooked, done].

If cooking is involved; Frying (deep-frying, frying, sautéing), grilling, roasting, baking, steaming, hot liquid (boiling, poaching, simmering, blanching, braising), stewing, chilling, freezing.

NO.	UNIT LOCATION	CATEGORY OF STREET FOOD	NAME OF STREET FOOD	PREPARATION METHOD	FREQUENCY	REMARKS
1						
2						
3						
4						
5						
6						
7						
8						
9						

TABLE S2 : EXPLANATION OF EACH STREET FOOD

Table S3: Description of 35 most frequently available street foods under the main meals category.

No.	Name of street food	Description of street food
1	Fried noodle	Yellow noodles that are stir-fried in sauces such as soy sauce, thick soy sauce, oyster sauce, and chili sauce. This dish is sometimes eaten with pickled chili.
2	*Kolo mee*	A signature dish from Sarawak. It is made with egg noodles that are tossed in sweet and salty soy sauce and served with plain soup.
3	Beef burger	A Malaysian burger with a ready-made beef burger patty that is grilled with butter, and placed in between two buttered buns. The patty is then topped with sauces such as chili sauce, mayonnaise, and seasoning sauce.
4	Kebab	Pita bread with grilled chicken fillings, and topped with chili sauce, mayonnaise, and seasoning sauce.
5	*Bakso*	A signature dish from Indonesia. 'Bakso' refers to meatballs that are made of beef surimi. This dish comprises any type of noodles served in a soup with beef meatballs that are cooked in beef stock. This dish is sometimes eaten with a soy sauce sambal.
6	Chicken burger	A Malaysian burger that comprises a ready-made chicken burger patty that is grilled with butter, and placed in between two buttered buns. The patty is then topped with sauces such as chili sauce, mayonnaise, and seasoning sauce.
7	Fried *kuey teow*	Flat rice noodles that are stir-fried in sauces such as soy sauce, thick soy sauce, and oyster sauce. This dish is sometimes eaten with pickled chili.
9	Noodle soup	Boiled yellow noodles served in chicken or beef-based clear soup. This dish is sometimes eaten with a soy sauce sambal.
8	*Char kuey teow*	A dish that is similar to fried kuey teow, except that it is not stir-fried until dry. This dish is sometimes eaten with pickled chili.
10	*Roti john*	An omelet sandwich that comprises an oblong-shaped bread that is sliced halfway in the middle, cooked on an omelet, and topped with sauces such as chili sauce, mayonnaise, and seasoning sauce.
11	Fried vermicelli @ fried *mihun*	Rice vermicelli stir-fried in sauces such as soy sauce, thick soy sauce, oyster sauce, and chili sauce. This dish is sometimes eaten with chili sauce.
12	*Soto*	A signature dish from Indonesia that comprises boiled cube rice served in a meat-based soup. This dish is sometimes eaten with a soy sauce sambal.

13	Fried rice	Overnight rice that is stir-fried with sauces such as soy sauce and oyster sauce. This dish is sometimes eaten with a soy sauce sambal.
14	Vermicelli soup @ *mihun sup*	Rice vermicelli served in a chicken or beef-based clear soup. This dish is sometimes eaten with a soy sauce sambal.
15	*Nasi lemak* with fried chicken	A Malay dish comprising fragrant rice cooked in coconut milk and pandan leaves. The rice is served with sambal (a mixture of chili paste, shrimp paste, garlic, ginger, and shallot), toasted peanuts, deep-fried anchovies, cucumber, and deep-fried chicken marinated with spices.
16	*Nasi tomato*	Rice cooked with tomato paste, ghee, evaporated milk, margarine, chicken cube, garlic, ginger, and shallots. The rice is served with *ayam masak merah* (chicken cooked in chili paste, chili sauce, and oyster sauce), and pickled pineapple and cucumber.
17	Noodles with gravy (curry or soy sauce)	Noodles served in coconut-based curry or tossed with a sauce mixture (thick soy sauce and oyster sauce).
18	*Nasi lemak*	A Malay dish comprising fragrant rice cooked in coconut milk and pandan leaves. The rice is served with sambal (a mixture of chili paste, shrimp paste, garlic, ginger, and shallot), toasted peanuts, deep-fried anchovies, cucumber, and hard-boiled egg.
19	*Kuey teow* soup	Flat rice noodles served in a chicken or beef-based clear soup. This dish is sometimes eaten with a soy sauce sambal.
20	*Laksa* (Penang style)	A signature dish from Penang comprising rice-based noodles served in a sour and spicy fish-based gravy and shrimp paste. As compared to *Laksa* (Perak style), the gravy is thinner and *kantan* flower is used instead of lemongrass.
21	*Nasi minyak*	Rice cooked with ghee and spices. The rice is often eaten with *ayam masak merah*.
22	*Nasi kerabu*	A signature dish from Kelantan that comprises blue-colored rice, often eaten with dried fish, fried chicken, or smoked beef. The dish is also served with crackers, salted eggs, raw vegetables, and sambal.
23	Chicken rice	A dish comprising yellow-colored seasoned rice that is served with roasted or poached chicken, cucumber, sambal, light soy sauce, and clear soup.
24	*Laksa* (Perak style)	A signature dish from Perak that comprises rice-based noodles served in a sour and spicy fish-based gravy and shrimp paste. As compared to *Laksa* (Penang style), the gravy is thicker and lemongrass is used instead of *kantan* flower.
25	Glutinous rice with *rendang* (meat cooked with spices)	A Malay dish comprising glutinous rice that is steamed with coconut milk, salt, and sugar, and served with either chicken or beef cooked with spices.

26	Chicken porridge	Rice that is cooked in boiling water until softened to a porridge consistency. Chicken that is marinated with oyster sauce is then added to the softened rice until cooked. Chicken or vegetable cube is often added to the porridge to add more flavor.
27	*Roti canai* (flat bread)	An Indian flatbread that is often served with *dhal* (lentil curry), fish curry, and sambal.
28	Rice porridge	Rice that is cooked in boiling water until softened to a porridge consistency. The porridge is then served with garnishes such as green onions, fried anchovies, and light soy sauce. Chicken or vegetable cube is often added to the porridge to add more flavor.
29	Net crepes (*Roti jala*)	A Malay dish made of a thin plain batter that is pan-fried without oil. The batter is poured into a funnel and onto a pan, to create a 'net.' This dish is eaten with meat curry.
30	Glutinous rice with fried fish	Steamed glutinous rice served with salted shredded coconut and fried salted mackerel.
31	Spaghetti bolognese	An Italian dish comprising spaghetti that is cooked in a minced-meat tomato-based pasta sauce.
32	*Laksam*	A signature dish from Terengganu that comprises flattened rice-based noodles served in a white-colored gravy (a mixture of fish, coconut milk, herb leaves, and shrimp paste).
33	Cubed rice with peanut gravy *(nasi impit)*	Cube-shaped rice cooked in boiling water and served with savory and spicy peanut gravy.
34	*Putu*	A signature dish from Sabah that comprises shredded tapioca that is shaped into a sausage and then steamed. This dish does not have any flavor and is eaten like rice, with seafood such as grilled fish.
35	*Sianglag*	A signature dish from Sabah. It is almost similar to *putu*, except that *sianglag* is not shaped and is pan-fried without oil. This dish does not have any flavor and is eaten like rice, with seafood such as grilled fish.

Table 2: Description of 30 most frequently available street foods under the snacks category in Malaysia.

No.	Name of street food	Description of street food
1	Fried fish ball	A mixture of processed fish meat with salt, sugar, monosodium glutamate (MSG), and chicken seasoning, made into balls, and then deep-fried.
2	*Keropok lekor*	Signature snack from Terengganu that is made from a mixture of sea fish flesh with sago flour, salt, sugar, MSG, and ice-cold water. The mixture is shaped into a sausage and then deep-fried. This snack can be eaten on its own or with chili sauce.
3	Seaweed pickle	Signature snack from Sabah that is made of seaweed or sea grapes. This snack can be eaten on its own, dipped in a sauce (chili, lime juice, and onion), or as a side dish with rice.
4	Fried chicken with cheese	Processed chicken that is added with cheese and then deep-fried.
5	Fried crab meatball	Crab meatball that is made of surimi, sugar, salt, MSG, and crab seasoning. The crab meatball is coated with breadcrumbs and then deep-fried.
6	Fried sausage	Processed chicken shaped into a sausage and then deep-fried.
7	Fried chicken ball	A mixture of processed chicken meat with salt, sugar, MSG, and chicken seasoning, made into balls and then deep-fried.
9	Fried sausage with cheese	Processed chicken shaped into a sausage, stuffed with cheese, and then deep-fried.
8	Chicken nuggets	A mixture of processed chicken meat with salt, sugar, and MSG. The nuggets are commonly shaped into hearts, coated with breadcrumbs, and then deep-fried.
10	*Kerepek*	Deep-fried savory crackers made of rice flour.
11	Takoyaki	A Japanese snack made of a wheat flour-based batter and cooked in a sphere molded pan. This snack is typically filled with minced or diced octopus and green onion. In Malaysia, this snack is garnished with bonito flakes and drenched with takoyaki sauce and mayonnaise.
12	Pizza	An Italian dish made of a flat, round wheat-based dough and is commonly topped with tomato sauced, cheese, and other toppings. The dough is then baked in a high-temperature oven along with the toppings.
13	Fried chicken	Chicken pieces coated in a seasoned batter and then deep-fried.
14	Fried chicken (non-meat parts)	Chicken pieces (e.g., gizzards) coated in a seasoned batter and then deep-fried.

15	Satay	Meat that is seasoned and skewered, and then grilled. This dish is often eaten with a savory and spicy peanut sauce, onion, and cucumber.
16	*Satar*	A signature dish from Terengganu that is made of fish flesh, shredded coconut, chili, onion, ginger, salt, sugar, and MSG. The mixture is wrapped in a banana leaf and grilled.
17	*Cakoi*	A plain Chinese snack made of wheat-based dough that is deep-fried.
18	Fried *popiah*	Deep-fried spring rolls with vegetable fillings. This snack can be eaten on its own or with sambal. The sambal sometimes contains dried shrimp.
19	*Pulut panggang*	A Malay dish made of steamed and salted glutinous rice, and topped with spiced shredded coconut fillings. The rice and fillings are wrapped in banana leaves and cooked by grilling.
20	Curry puff (potato)	A small deep-fried pie with potato curry fillings.
21	*Murtabak*	A square-shaped, pan-fried pancake that is stuffed with minced meat, eggs, garlic, and onion.
22	*Jering rebus*	Signature snacks from Kelantan that are made of boiled stinky beans. This snack is eaten with seasoned shredded coconut.
23	French fries	Deep-fried potato strips. This snack is often sprinkled with salt.
24	*Kuih cara berlauk ayam*	A Malay savory traditional cake made of a wheat flour-based thin batter and cooked in a molded pan. The cooked batter is then topped with a mixture of minced chicken, curry powder, onion, garlic, and white or black pepper.
25	*Kuih tepung gomak*	A snack made of glutinous rice-based dough, with shredded coconut fillings, and cooked in boiling water. The cooked snack is then coated with fried mung bean flour.
26	Grilled chicken (small pieces)	Chicken pieces that are marinated and grilled.
27	Corn (savory)	Grilled corn on the cob that is brushed with margarine and salt.
28	Egg tart	A snack made of oven-baked pastry crust with egg custard fillings.
29	*Chee cheong fun*	A Chinese dish made of steamed rice noodle rolls served with soy sauce and garnished with fried onions.
30	*Beh hua chee*	A Chinese deep-fried dish made of wheat-based dough.

Table 3: Description of 29 most frequently available street foods under the desserts category.

No.	Name of street food	Description of street food
1	*Apam balik* with cheese	A sweet pancake that is filled with peanuts, sugar, corn, margarine, and shredded cheese.
2	*Kuih kacang*	A Malay traditional cake made of a mixture of mung beans, coconut, and sugar, and dipped in a thin batter. The mixture is then deep-fried.
3	*Putu piring*	Round-shaped, steamed rice flour, filled with palm sugar.
4	*Kuih bom*	A Chinese traditional cake made by deep-frying a glutinous rice flour batter, filled with coconut fillings, and coated with sesame.
5	Banana fritters with cheese	A banana that is coated in a batter and deep-fried. The fritters are then topped with shredded cheese.
6	*Kuih seri muka*	A Malay traditional cake that comprises a layer of steamed glutinous rice and another layer of green custard made from pandan juice.
7	*Apam balik telur*	A signature dish from Penang, made of a thin pancake and served with shredded coconut.
9	*Kuih akok*	A traditionally baked cake from the East Coast of Malaysia, made of a mixture of egg, wheat flour, coconut milk, salt, water, and palm sugar.
8	Donut	Deep-fried leavened dough, coated with caster sugar.
10	*Apam balik*	A sweet pancake that is filled with peanuts, sugar, corn, and margarine.
11	*Kuih puteri ayu*	A traditional Malay cake made by steaming a thin batter consisting of wheat flour, coconut milk, pandan juice, and corn flour, on top of salted shredded coconut in a molded pan.
12	Banana fritters	A banana that is coated in a batter and deep-fried.
13	*Kuih sagu*	A traditional cake made by steaming sago pearls that are boiled with pandan leaves, sugar, and rose syrup. The steamed cake is then coated with salted shredded coconut.
14	*Kuih cek mek molek*	A traditional cake from Kelantan, made of deep-frying mashed sweet potato filled with sugar.
15	Popcorn	Corn kernels that are heated to 'pop' or expand, and coated in caramel sauce.
16	*Kuih keria*	A traditional cake that is made by deep-frying a mixture of mashed sweet potatoes and wheat flour. The fried cake is then coated with

		sugar syrup.
17	*Kuih lepat*	A Malay traditional cake made by steaming a mixture of mashed bananas, sugar, wheat flour, rice flour, salt, and shredded coconut, and wrapping it in banana leaves.
18	*Kuih calak kuda*	A traditional cake from Sabah, that is made of wheat flour-based batter. The batter is baked in an oven and then layered with a chocolate spread. The cake is then folded into a horseshoe shape.
19	*Kuih buah Melaka*	Boiled rice cake balls filled with molten palm sugar. The cooked rice cake balls are then coated with salted shredded coconut.
20	*Kuih lapis*	A steamed colorful layered traditional cake made from a thin batter mixture of rice flour, wheat flour, corn flour, coconut milk, sugar, salt, and pandan leaves.
21	*Cekodok*	A deep-fried batter made of overripe bananas, wheat flour, salt, and sugar.
22	Steamed baozi with sweet fillings	Chinese steamed buns with various types of sweet fillings such as red beans, shredded coconut, or coconut jam.
23	*Peneram*	A traditional cake made from a dough mixture of rice flour, palm sugar, and water. The dough is then cooked by deep-frying.
24	*Cendol*	An iced sweet dessert that comprises green rice flour jelly and red beans, served in salted coconut milk and topped with palm sugar syrup.
25	*Kuih apam*	A Malay traditional cake made from a dough mixture of rice flour, wheat flour, yeast, sugar, water, and blended rice. The dough is cooked by steaming and topped with salted shredded coconut.
26	*Kuih jelurut*	A traditional cake made from a thin batter mixture of rice flour, sugar, salt, and coconut milk, that is filled in a cone-shaped coconut leaf and steamed.
27	*Tau fu fa*	A Chinese traditional dessert made of silken smooth tofu served in sugar syrup. This dessert can be eaten either warmed or chilled.
28	*Kuih penjaram*	A sweet traditional cake from Sabah, made of wheat flour and rice flour batter, sugar, and salt. The batter is cooked by deep-frying.
29	*Kuih angku*	A Chinese traditional cake made of glutinous rice flour skin with sweet shredded coconut or mung bean fillings. The cake is molded into a small round shape, placed on a banana leaf, and steamed.
